# Gp130-dependent STAT3 activation in M–CSF–derived macrophages exaggerates tumor progression

**DOI:** 10.1016/j.gendis.2023.05.004

**Published:** 2023-06-20

**Authors:** Yuting Xu, Xiaoyu Xu, Qianyue Zhang, Peizhe Lu, Chongjun Xiang, Lei Zhang, Chunhua Lin, Qiaoling Song

**Affiliations:** a Key Laboratory of Marine Drugs, Ministry of Education of China, School of Medicine and Pharmacy, Ocean University of China, Qingdao, Shandong 266003, China; b College of Marine Life Sciences, Ocean University of China, Qingdao, Shandong 266071, China; c Department of Neuroscience, University of Michigan, Ann Arbor, MI 48103, USA; d Department of Urology, The Affiliated Yantai Yuhuangding Hospital of Qingdao University, Yantai, Shandong 264000, China; e Research Center of Traditional Chinese Medicine and Clinical Pharmacy, Shandong Provincial Maternal and Child Health Care Hospital Affiliated to Qingdao University, Jinan, Shandong 250014, China; f Innovation Platform of Marine Drug Screening & Evaluation, Qingdao National Laboratory for Marine Science and Technology, Qingdao, Shandong 266100, China

Interleukin-6 (IL-6) is a common pluripotent cytokine that is highly expressed in the tumor microenvironment. The IL-6/JAK/STAT3 signaling directly promotes tumor progression, severely hampering antitumor immunity. Macrophages, the richest innate immune cells within the tumor microenvironment, manipulate tumor progression via phenotypic plasticity. Granulocyte-macrophage colony-stimulating factor (GM-CSF) and macrophage colony-stimulating factor (M-CSF) are two critical growth factors regulating macrophage differentiation and function. They play different roles during tumor development; while GM-CSF links with enhanced antitumor immunity, M-CSF is associated with M2-like phenotypes of tumor-associated macrophages. Nevertheless, the signaling response of differentially differentiated macrophages to IL-6 has not yet been elucidated. Here, we explored IL-6 downstream signaling events in two distinct macrophage populations (M1-like proinflammatory GM-CSF primed and M2-like immunosuppressive M-CSF primed bone marrow-derived macrophages, GM-BMDMs *vs*. M-BMDMs). We found that IL-6 preferentially induces JAK2/TYK2/STAT3 activation in M-BMDMs due to their higher glycoprotein 130 (gp130) expression. *In vivo*, STAT3 phosphorylation is largely present in macrophage populations within the tumor microenvironment. Importantly, the gp130 inhibitor could effectively reverse the tumor-promoting effects of M-BMDMs via IL-6/STAT3 signaling blockage, pointing to its potential therapeutic usage for tumor treatment.

In our current study, STAT3 was highly activated in M-BMDMs post IL-6 treatment (Fig. 1A; Fig. S1A) and the phosphorylation could sustain as long as 48 h (Fig. S1B). Phosphorylation of upstream regulators JAK2 and TYK2, but not JAK1, was responsible (Fig. 1A). No other STAT family members' activation was observed post IL-6 treatment (Fig. S1C). Transient activation of the MAPK pathway (ERK, p38, and JNK) was observed post IL-6 challenge but no differences were observed (Fig. S1D). Next, expression levels of JAKs and STATs in these two macrophage populations were determined. No observed overexpression of STAT3 and JAKs in M-BMDMs did account for IL-6-induced STAT3 activation in M-BMDMs (Fig. 1B; Fig. S2A). Classically, IL-6 binds to IL-6R and recruits gp130 to form IL-6/IL-6R/gp130 heterotrimer to initiate STAT3 signaling. The expression levels of IL-6R (Fig. S2B) and gp130 (Fig. 1C; Fig. S2C) were significantly higher in M-BMDMs, indicating that the insufficient expression of these receptors might account for less STAT3 activation in GM-BMDMs. Interestingly, the exogenous addition of overdose IL-6R failed to induce STAT3 phosphorylation in GM-BMDMs (Fig. S2D). Another IL-6 family cytokine LIF, which depends on gp130 as its signaling receptor β subunit but not IL-6R, could also induce higher levels of STAT3 activation in M-BMDMs (Fig. 1D), pointing to the essential role of gp130 for STAT3 activation. All these data together suggest that the stronger STAT3 activation in M-BMDMs in response to IL-6 family members results from higher gp130 expression.

**Figure 1:**
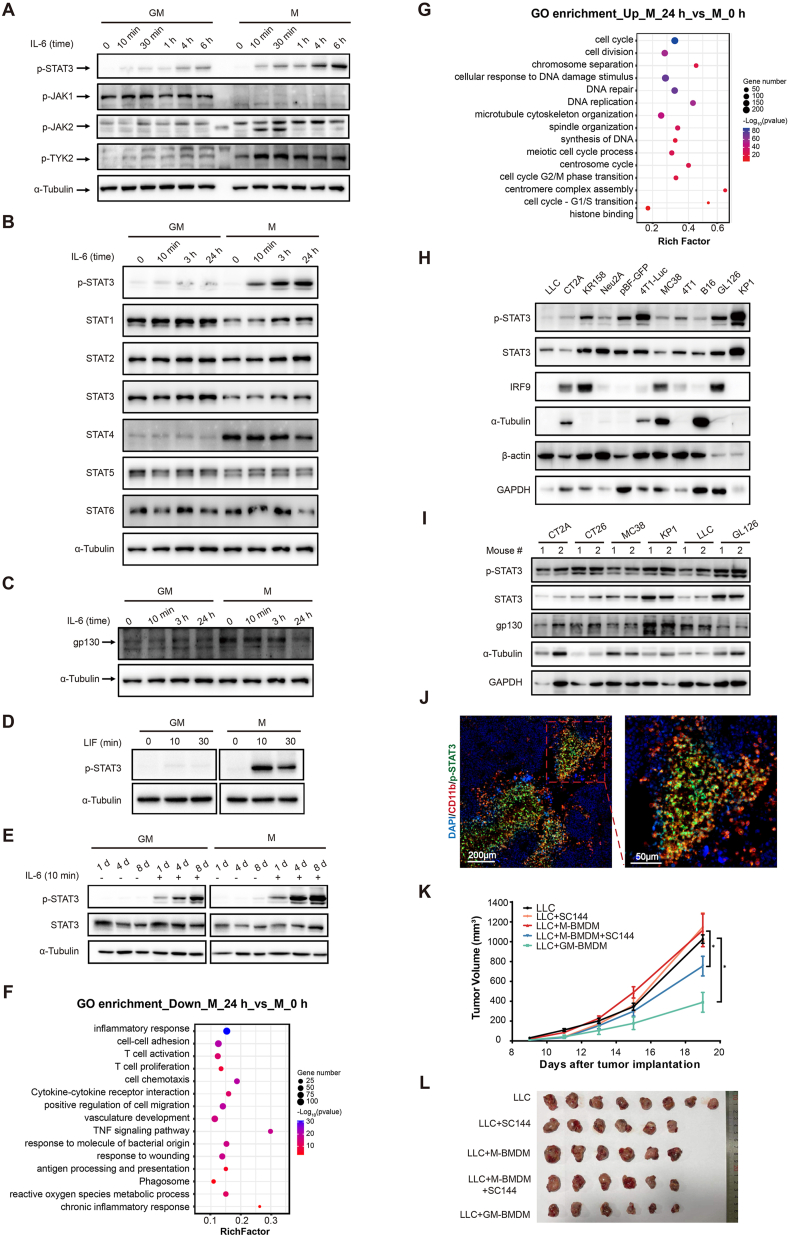
The IL-6/gp130/STAT3 signaling affected the function of M-BMDMs and significantly promoted tumor growth *in vivo*. **(A**–**C)** GM-BMDMs and M-BMDMs were treated with IL-6 (20 ng/mL) for indicated times. Whole-cell lysates were processed for Western blot analysis and probed with antibodies against pTyr705-STAT3, pTyr1022/1023-JAK1, pTyr1007/1008-JAK2, and pTyr1054/1055-TYK2 for (A), anti-pTyr705-STAT3, anti-STAT1, anti-STAT2, anti-STAT3, anti-STAT4, anti-STAT5, and anti-STAT6 for (B), and gp130 for (C). STAT5 antibodies in (B) recognize endogenous levels of the total STAT5 protein (STAT5a and STAT5b). Anti-α-Tubulin antibodies were used as loading controls. **(D)** GM-BMDMs and M-BMDMs were cultured and treated with LIF (30 ng/mL) for indicated times. Whole-cell lysates were processed for Western blot analysis and probed with anti-pTyr705-STAT3 antibody. Anti-α-Tubulin antibodies were used as loading controls in all the above-described experiments. **(E)** The bone marrow cells were isolated and treated with GM-CSF or M-CSF for a series of periods (1 d, 4 d, 8 d), followed by stimulation with or without 20 ng/mL IL-6 for 10 min. The expression level of STAT3 Y705 phosphorylation was detected using Western blot analysis. **(F, G)** The Gene Ontology analysis of down-regulated (F) or up-regulated DEGs (G) between M 24 h and M 0 h. GM-BMDMs and M-BMDMs were cultured and treated with IL-6 (20 ng/mL) for 3 h (M 3 h) or 24 h (M 24 h) or without IL-6 (M 0 h). Cells were harvested after treatment and their RNA was used for RNA-seq. “Rich factor” represents the proportion of DEGs to all the genes that are annotated in a specific pathway term. The greater the rich factor, the greater the degree of enrichment. **(H)** Different types of mouse tumor cells were lysed for protein extraction and subjected to Western blot analysis using anti-pTyr705-STAT3, anti-STAT3, and anti-IRF9 antibodies. Anti-α-Tubulin, anti-β-actin, and anti-GAPDH antibodies were used as loading controls. **(I)** The various types of tumor tissues stripped from subcutaneous tumor-bearing mice were dissociated and collected for protein extraction, followed by Western blot analysis using anti-pTyr705-STAT3, anti-STAT3, and anti-gp130 antibodies. Anti-α-Tubulin and anti-GAPDH antibodies were used as loading controls. **(J)** Double-color immunofluorescence co-localization of p-STAT3 (green) and CD11b (red) in LLC cell-derived subcutaneous mouse tumor tissue. Original magnification = 200×. **(K, L)** LLC subcutaneous tumor-bearing mice injected by macrophages (GM-BMDMs/M-BMDMs) or vehicle were treated with SC144 (5 mg/kg, intraperitoneally, once daily) or vehicle for 3 days. The tumor volumes were calculated (K). Tumors were excised and photographed after sacrificing (L). Statistical significance was calculated by two-way ANOVA for comparisons in (K). Significant differences are indicated as ∗*P* < 0.05.

Next, we determined at which time points of macrophage differentiation the different responses to IL-6 occurred. The freshly isolated bone marrow cells were stimulated with GM-CSF or M-CSF for indicated times followed by vehicle or IL-6 stimulation. The STAT3 phosphorylation levels were not significantly elevated until 4 days of GM-CSF/M-CSF incubation (Fig. 1E; Fig. S3A, B). This suggests that IL-6-induced STAT3 activation requires long-lasting M-CSF priming instead of a transient immediate stimulation. In addition, significantly higher STAT3 phosphorylation levels in the G>M group (6-day incubation with GM-CSF followed by 1-day M-CSF treatment) than in the G>G control group (continuous 7-day GM-CSF incubation) post IL-6 challenge (Fig. S3C), pointing to the direct effects of M-CSF for the strong STAT3 activation. These data suggested the relatively long-term training by M-CSF is directly responsible for the activated IL-6/gp130/STAT3 cascade in BMDMs.

Transcriptome analysis was performed to systemically elucidate the influence of IL-6-mediated STAT3 activation for M-BMDMs. Twenty-four hours of IL-6 treatment changes numerous gene expressions over 3 h of treatment (Fig. S4A, B). Functional enrichment analysis (*M*_24 h *vs*. M_0 h) revealed multiple tumor-suppressive effects of macrophages such as phagosome, antigen processing and presentation, and T cell activation were down-regulated (Fig. 1F). The related genes such as Tnf, Ccl2, Tlr2, and H2-M2 were cored in down-regulated protein–protein interaction networks (Fig. S5A). Besides, the function of the lysosome, the major organelle for phagocytosis, was inhabited as early as 3-h IL-6 treatment (Fig. S5B). To validate these findings, LLC (Lewis lung carcinoma) cells were co-cultured with M-BMDMs, and the phagocytic activity of M-BMDMs towards LLC cells decreased after IL-6 treatment (Fig. S5C, D). Meanwhile, canonical STAT3 downstream signaling pathways such as cell cycle, cell division, and DNA replication were up-regulated by IL-6 treatment (Fig. 1G), which were further verified by ingenuity pathway analysis (Fig. S4C) and protein–protein interaction networks (Fig. S4D).

Survival analysis revealed that cancer patients with higher IL-6 expression had a worse prognosis (Fig. S6A), pointing to the strong relevance of IL-6 signaling with tumor growth. *In vitro*, STAT3 activation and IL-6 mRNA levels varied greatly among different cancer cell lines (Fig. 1H; Fig. S5B). Interestingly, STAT3 phosphorylation and gp130 expression were generally high in mouse tumor tissues *in vivo*, regardless of tumor cell types (Fig. 1I). Meanwhile, mRNA levels of gp130-dependent IL-6 superfamily cytokines were highly expressed in most subcutaneous tumors (Fig. S6C–E). In particular, IL-6 mRNA levels in subcutaneous tumors were inconsistent with their expression levels in tumor cells (Fig. S6B), indicating that IL-6 expression *in vivo* is not dependent on cancer cells. Further flow cytometry analysis revealed the presence of a large number of CD11b^+^ myeloid leukocytes within the LLC tumor microenvironment, of which CD11b^+^ F4/80^+^ CD206^+^ M2 macrophages accounted for approximately 70% (Fig. S6F). Besides, STAT3 phosphorylation was mainly detected in CD11b^+^ cells (Fig. 1J). These data implied that M2-like macrophages could be a significant contributor to high STAT3 activation within the tumor microenvironment *in vivo*.

Therapeutically, gp130 blocker SC144 was added to investigate the contribution of IL-6/STAT3 signaling to tumor progression in subcutaneous LLC and BMDM co-implantation models *in vivo* (Fig. S7A). Consistent with macrophage characteristics, co-injected M-BMDMs provoke and GM-BMDMs suppressed LLC tumor growth (Fig. 1K, L; Fig. S7C). SC144 did significantly reverse M-BMDM-promoted tumor growth with no observed inhibitory effects for LLC alone group (Fig. 1K, L; Fig. S7C), indicating that early SC144 treatment suppresses tumor growth mainly by interfering with IL-6/gp130/STAT3 cascade in M2 macrophages, but not in tumor cells. No obvious drug toxicity or weight loss was observed post SC144 treatment (Fig. S7B).

The hyper-activation of the IL-6/JAK/STAT3 cascade plays a bi-directional role in tumor progression and the related therapeutic strategies to target this pathway failed in a panel of tumor types.^1^^,^^2^ To date, studies for IL-6-mediated STAT3 activation within the tumor microenvironment mainly focus on tumor cells, T cells, and DC cells. Only limited studies reported STAT3 activation in macrophages and few explored the preference of STAT3 activation in different types of macrophages. The innate antitumor immunity was increased in the hematopoietic conditional STAT3 knockout mice.^3^ Macrophage conditional STAT3 knockout mice increased expressions of pro-inflammatory mediators.^4^ Blocking STAT3 activation inhibits tumor-associated macrophages' polarization to M2 phenotype.^5^ These findings prompted us to investigate the differences in response to IL-6 of the two distinct types of macrophages. Our research provides new insight into the IL-6/STAT3 signaling regulation in distinct macrophage populations and further proves the therapeutic potential of gp130 blockage for tumor treatment. We acknowledge that our study has limitations as we only investigated one cell line model. Further research involving multiple cell line types would enhance the clarity and robustness of our findings. Since STAT3 is highly activated in macrophages within the tumor microenvironment, combinational treatment of anti-gp130 and anti-PD-1 for T cell re-activation, or targeted drug for tumor cells, could be an optimal regimen for cancer treatment, avoiding the dilemma of insufficient elimination of tumors by STAT3 monotherapy.

## Author contributions

All authors participated in interpreting the whole data. Q.S. designed and supervised the study; L.Z., Y.X., X.X., Q.Z., and C.X. performed and evaluated individual experiments; Y.X. and Q.S. performed bioinformatical analyses; Y. X, L.Z., P.L., and C.L. wrote the manuscript with the contributions from all the authors.

## Conflict of interests

The authors declare no conflict of interests.

## Funding

This work was financially supported by the Shandong Province Major Scientific and Technological Innovation Project (China) (No. 2020CXGC010503), the Shandong Provincial Key Laboratory Platform Project (China) (No. 2021ZDSYS11), and the 10.13039/501100001809National Natural Science Foundation of China Major Project (No. 81991525).

## Figures and Tables

**Fig. S1:**
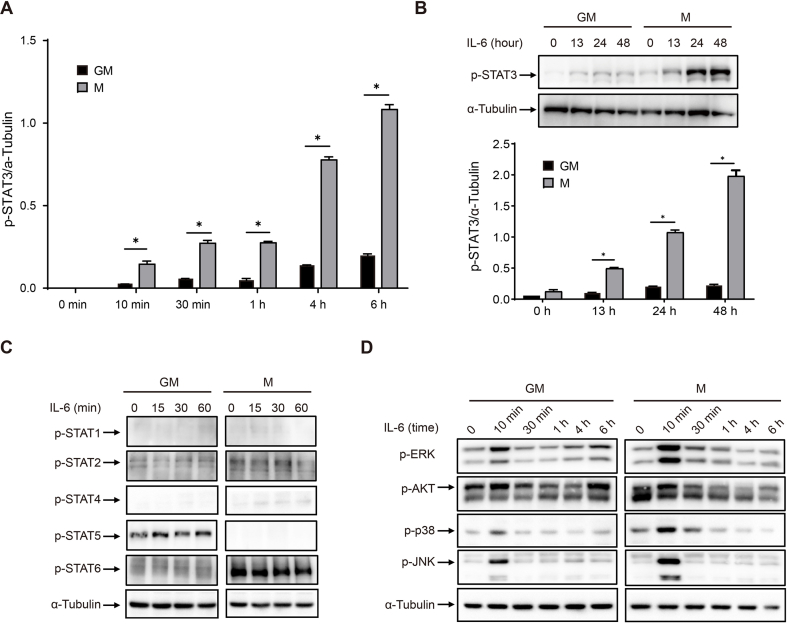
Figure S1 IL-6 specifically induced JAK2/TYK2/STAT3 activation in M-BMDMs. (A) The intensity of the p-STAT3 bands in Figure 1A was quantified. (B-D) GM-BMDMs and M-BMDMs were treated with IL-6 (20 ng/ml) for the indicated times. Whole cell lysates were processed for Western blot analysis and probed with antibodies against pTyr705-STAT3 for (B), pTyr701-STAT1, pTyr690-STAT2, pTyr693-STAT4, pTyr694-STAT5 and pTyr641-STAT6 for (C) and pThr202/Tyr204-ERK, pThr308-AKT, pThr180/Tyr182-p38, and pThr183/Tyr185-JNK for (D). pThr202/Tyr204-ERK antibodies in (C) detect endogenous levels of total p44/42 MAP kinase (Erk1/Erk2) protein. Anti-α-Tubulin antibodies were used as loading controls. The intensity of the p-STAT3 bands in (A, B) was quantified using ImageJ.

**Fig. S2:**
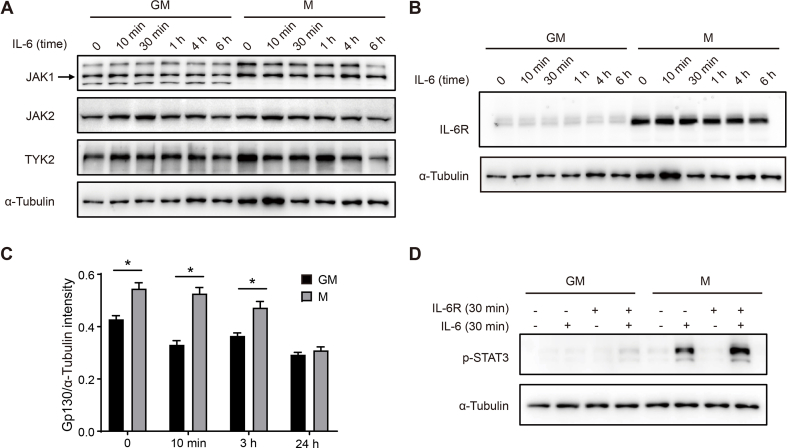
Figure S2 STAT3 activation in M-BMDMs post IL-6 challenge might result from the high expression of gp130 rather than IL6R. (A-B) GM-BMDMs and M-BMDMs were cultured and treated with IL-6 (20 ng/ml) for the indicated times. Whole cell lysates were processed for Western blot analysis and probed with antibodies including anti-JAK1, anti-JAK2 and anti-TYK2 for (A) and anti-IL-6R for (B). (C)The intensity of the gp130 bands was quantified using ImageJ. (D) GM-BMDM and M-BMDM were cultured and treated with or without IL-6 for 30 min in the presence or absence of IL-6R (10 ng/ml) and the expression level of STAT3 Y705 phosphorylation was detected using Western blot analysis.

**Fig. S3:**
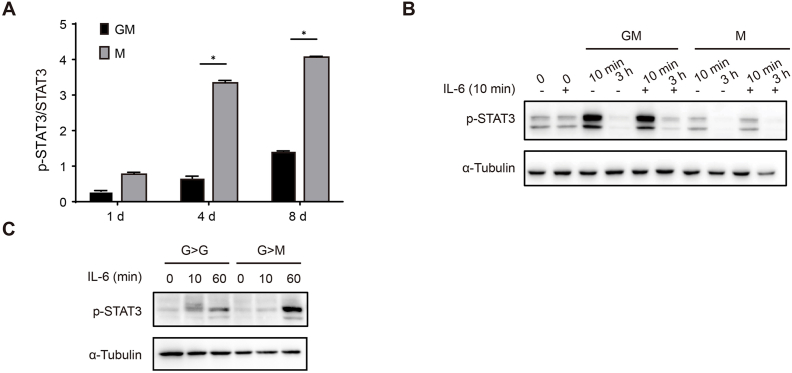
Figure S3 M-CSF could induce strong activation of STAT3 to reverse the inhibitory effects of GM-CSF. (A) The intensity of the p-STAT3 bands in Figure 1E was quantified using ImageJ. (B) The BMs were isolated and treated with GM-CSF or M-CSF for a series of time periods (0, 10 min, 3 h), followed by stimulation with or without 20 ng/ml IL-6 for 10 min. The expression level of STAT3 Y705 phosphorylation was detected using Western blot analysis. (C) Following 6 days treatment with GM-CSF, the BMs were then continuously cultured for another 24 h with GM-CSF or M-CSF instead of GM-CSF. The expression level of STAT3 Y705 phosphorylation was measured after stimulation with IL-6 with the indicated times. Anti-α-Tubulin antibodies were used as loading controls in all of the above-described experiments.

**Fig. S4:**
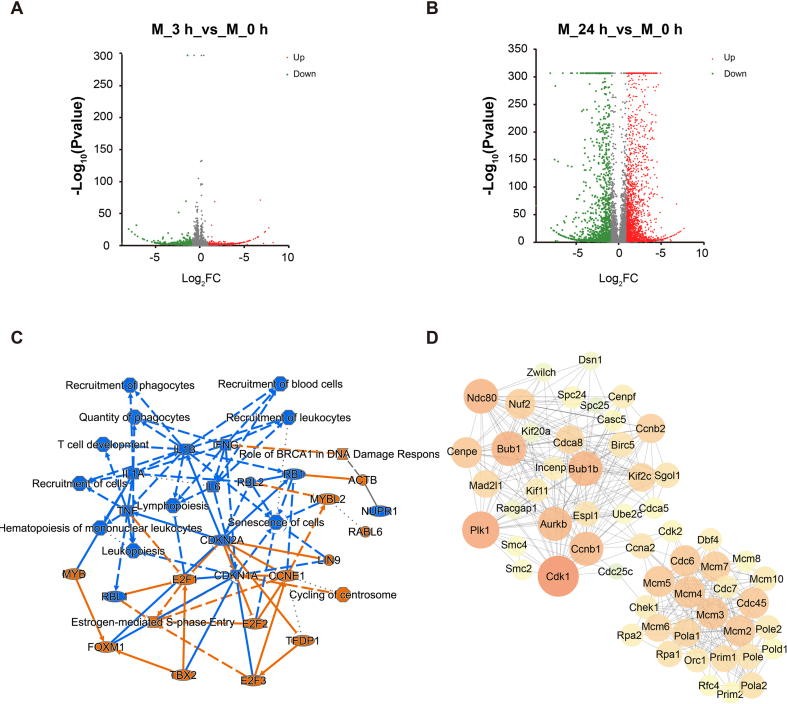
Figure S4 IL-6 induced transcriptional activation of cell cycle-related genes in M-BMDMs. (A-B) GM-BMDMs and M-BMDMs were cultured and treated with IL-6 (20 ng/ml) for 3 h (M 3 h) or 24 h (M 24 h) or without IL-6 (M 0 h). Cells were harvested after treatment and their RNA were used for RNA-seq. Volcano plots showing fold change and p value for M 3 h compared with M 0 h (A) and M 24 h compared with M 0 h (B). Up-regulated DEGs are depicted in red, and down-regulated DEGs are depicted in green. (C) The graphical summary of the significant difference between M 24 h and M 0 h groups by IPA analysis. Symbols of target proteins, canonical signaling, immune cell regulation and diseases in blue and orange indicate a predicted downregulation and up regulation, respectively. (D) PPI network analysis of up-regulated DEGs between M 24 h and M 0 h groups. Each node represents a protein and each edge represents an interaction between proteins. The depth of the color and the size of the node represents the degree of enrichment, the deeper the color and the larger the size represents the higher the degree of enrichment.

**Fig. S5:**
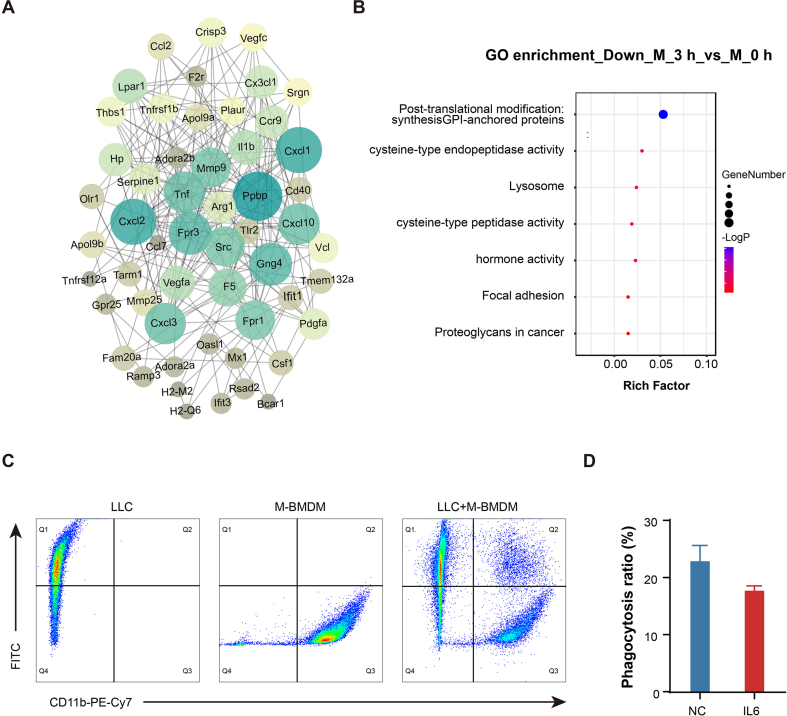
Figure S5 IL-6 induced down-regulation of inflammatory-related functions and phagocytosis in M-BMDMs. (A) PPI network analysis of down-regulated DEGs between M 24 h and M 0 h groups. (B) Down-regulated DEGs between M 3 h and M 0 h. “Rich factor” represents the proportion of DEGs to all the genes that are annotated in a specific pathway term. The greater of the Rich factor, the greater the degree of enrichment. (C) Flow cytometry analysis of the indicated co-cultures of M-BMDMs and LLCs. LLCs were incubated in advance with CellTracker TM (1:1000 dilution) staining solution (green) for 30 min at 37 °C and protected from light. Afterwards, LLCs (1 x 106) and M-BMDMs (2 x 105) were co-incubated for 6 hours with or without IL-6 stimulation, followed by flow analysis, and M-BMDM was labeled with anti-CD11b-PE-Cy7. (D) Bar graph shows quantification of flow cytometry analyses in (C). Phagocytosis ratio  = Cells in Q2/(Q2+Q3).

**Fig. S6:**
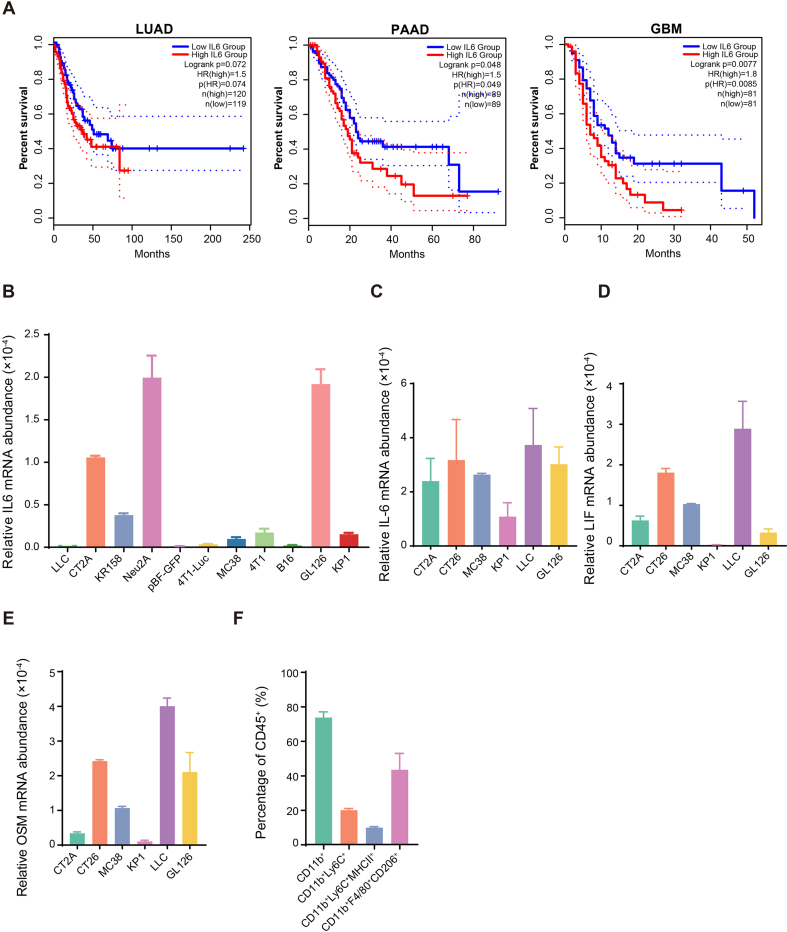
Figure S6 In TME, macrophage could be a significant contributor to high STAT3 activation. (A) Survival analysis of IL-6 expression in different tumor types (based on TCGA data in GEPIA). The red line represents samples with high gene expression and the blue line represents samples with low gene expression. Dotted line indicates 95% confidence interval, HR: hazard ratio. (B) Different types of mouse tumor cells were collected for RNA extraction and assessed by quantitative real-time PCR to detect the genes expression of IL-6. mRNA expression profiles are displayed as the fold increase of gene expression normalized to β-actin. (C-E) The various types of tumor tissues stripped from subcutaneous tumor-bearing mouse were dissociated and collected for RNA extraction and assessed by quantitative real-time PCR to detect the genes expression of IL-6, LIF and OSM. mRNA expression profiles are displayed as the fold increase of gene expression normalized to β-actin. (F) Flow cytometric analysis and quantification of macrophage infiltration in LLC cell-derived subcutaneous mouse tumor tissue.

**Fig. S7:**
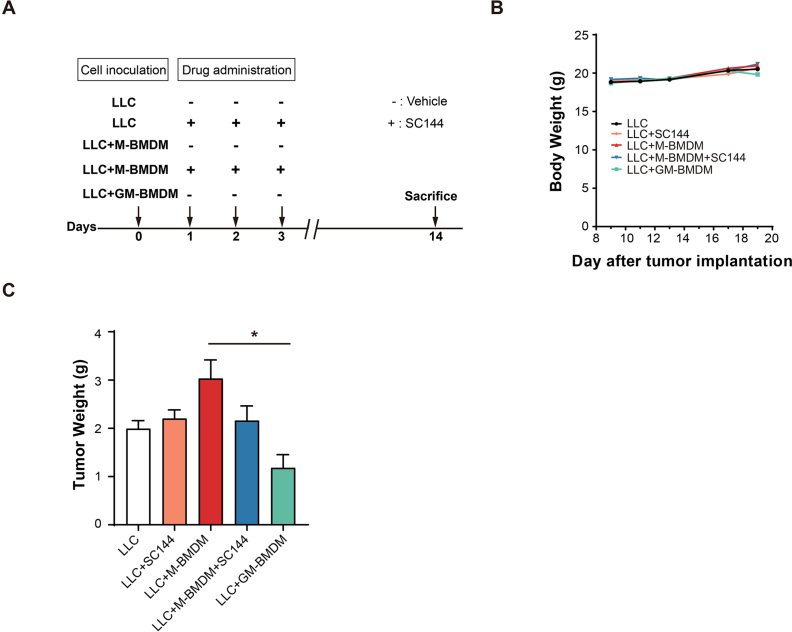
Figure S7 Gp130 blockage reversed M-BMDM dependent tumor progression. (A-C) LLC subcutaneous tumor-bearing mouses injected by macrophages (GM-BMDMs/M-BMDMs) or vehicle were treated with SC144 (5 mg/kg, i.p., q.d.) or vehicle for 3 days (A). The body weight changes (B) of mice were measured, and the tumor weight (C) were calculated.
