# Supplementary material for: Gp130-dependent STAT3 activation in M–CSF–derived macrophages exaggerates tumor progression
Source: Genes Dis. 2023 Jun 20;11(3):100985. doi: 10.1016/j.gendis.2023.05.004 (PMC10808918; doi:10.1016/j.gendis.2023.05.004)
Supplement: Multimedia component 1 [file mmc1.docx]

# Material and methods

## Antibodies and Reagents

In western blotting assay, primary antibodies for mouse STAT1 (Cat. 14994), STAT2 (Cat. 72604), STAT3 (Cat. 12640), STAT4 (Cat. 2653), STAT5 (Cat. 25656), STAT6 (Cat. 5397), JAK1 (Cat. 3332S), JAK2 (Cat. 3230), Tyk2 (Cat. 9312), p-Tyr701-STAT1 (Cat. 9167), p-Tyr690-STAT2 (Cat. 88410), p-Tyr705-STAT3 (Cat. 9145), p-Tyr693-STAT4 (Cat. 4134), p-Tyr694-STAT5 (Cat. 9359), p-Tyr641-STAT6 (Cat. 9361), p-Tyr1022/1023-JAK1 (Cat. 3331), p-Tyr1007/1008-JAK2 (Cat. 3776), p-Tyr1054/1055-Tyk2 (Cat. 9321), p-Ser473-AKT (Cat. 4060), p-Thr180/Tyr182-p38 MAPK (Cat. 4511), p-Thr183/Tyr185-SAPK/JNK (Cat. 4668), p-Thr202/Tyr204 MAPK (Erk1/2) (Cat. 4695), gp130 (Cat. 3732) and β-actin (Cat. 4970) were obtained from Cell Signaling Technology, antibodies against α-tubulin (Cat. sc-5286), IL-6R (Cat. sc-374259) were acquired from Santa Cruz.

For flow cytometry analysis, antibodies against CD11b (PE-Cy7) (Cat. 25-0112-82), CD45 (APC-Cy7) (Cat. 47-0451-82), CD8 (PerCP-eFluor™ 710) (Cat. 46-0081-82), CD19 (APC) (Cat. 17-0193-82), CD16/CD32 (Cat. 14-0161-86) and Rat IgG1 kappa isotype control (Cat. 14-4301-85) were from Invitrogen, antibodies against CD11b (FITC) (Cat. 130-113-805), CD69 (PE) (Cat. 104507), CD4 (FITC) (Cat. 100406), MHC Ⅱ (BV421) (Cat. 107632), F4/80 (PE) (Cat. 123110), CD206 (PE-Cy7) (Cat. 141720) were from Biolegend, antibodies against Ly6C (APC) (130-111-779) were from Miltenyi Biotec, and Fixable Viability Stain 510 (FVD) (Cat. 564406) was purchased from BD Biosciences.

Recombinant mouse M-CSF (Cat. 415-ML) and GM-CSF (Cat. 416-ML) were purchased from R&D Systems. Recombinant mouse IL-6 (Cat. 50136-MNAE) and mouse leukemia inhibitory factor (LIF) (Cat. AF-300-05) were from Peprotech. SC144 (Cat. T6207) were purchased from Shanghai Topscience Co., Ltd. SYBR Green PCR Master Mix (2 ×) (Cat. 4913914001), RNAiso Plus (Cat. 9109) and PrimeScript^TM^ RT reagent kit (Cat. RR047A) with gDNA eraser were from TaKaRa. BCA protein assay kit. 2,2,2-Tribromoethanol (Cat. T48402-5G) was obtained from Sigma-Aldrich. CellTracker^TM^ Green CMFDA Dye (Cat. C2925) was acquired from Invitrogen.

**Cell lines and Cell Culture**

LLC, CT2A, KR158, Neu2A, BF-GFP, 4T1-Luc, MC38, 4T1, B16, GL126, KP1 cells were cultured in Dulbecco’s modified Eagle’s medium (DMEM) supplemented with 10% fetal bovine serum (FBS), penicillin (100 IU/ml), and streptomycin (100 mg/ml), and the cells were maintained at 37 °C in an incubator containing 5% CO_2_.

**Mice and BMDM Culture**

Six weeks C57BL/6J mice (SPF degree) were obtained from Beijing Vital River Laboratory Animal Technology Co., Ltd. and maintained in a temperature- and humidity- controlled room with a 12-h light-dark cycle. Details of primary bone marrow cells (BMs) isolation have been reported previously [^36^](#_ENREF_36). After centrifugation and cell counting, BMs were resuspended in DMEM containing 10% FBS, 100 U/ml penicillin, 100 mg/ml streptomycin, either with 50 ng/ml M-CSF or 20 ng/ml GM-CSF. Cells (2 × 10^6^ for M-CSF or 4 × 10^6^ for GM-CSF) were seeded in a 60 mm dishes and cultured at 37℃ with 5% CO_2_ for 7 days. All animal experiments were approved by the Committee of Experimental Animals of Ocean University of China (OUCSMP-20191001).

**Western Blotting**

BMDMs were washed with cold PBS and harvested in lysis buffer containing protease and phosphatase inhibitors. A total of 20 μg protein lysates were resolved by SDS-PAGE electrophoresis gel and transferred onto nitrocellulose membranes (GE Healthcare, Cat. 10600034). After blocking with 5% nonfat milk solution, the membranes were probed with primary antibodies at 4℃ overnight and then incubated with horseradish peroxidase-conjugated secondary antibodies for 2 hours at room temperature. Immune complexes were detected with an Immobilon™ western chemiluminescence horseradish peroxidase substrate (Millipore, Cat. WBKLS0500) and photographed with a Tanon 5200 imaging system. The quantification was performed with ImageJ software.

**Transcriptome Sequencing**

BMDMs were treated with or without IL-6 (20 ng/ml) for 3 hours or 24 hours. Total RNA was prepared using RNAiso Plus. After the total RNA was isolated, cDNA library construction and sequencing were performed in a Illumina Novaseq6000 platform. Clean reads were aligned to the mouse reference genome using HISAT2. The expression levels for each of the genes were normalized to transcripts per kilobase million (TPM). Differential expression analysis was performed by DEGseq. Genes with the parameter of false discovery rate (FDR) below 0.001 and absolute fold change ≥2 were considered to be differentially expressed genes (DEGs). Hierarchical cluster analysis of DEGs was performed to explore gene expression patterns. GO enrichment analysis, Protein-protein interaction (PPI) and ingenuity pathway analysis (IPA) of DEGs were respectively performed. The RNA-seq data were deposited in the Gene Expression Omnibus (GEO) database under the accession number GSE179637.

**Flow Cytometry**

Cells were washed with PBS and resuspended in blocking buffer (PBS containing 20% FBS, 1:100 CD16/CD32 and 1:100 Rat IgG1 isotype) for 30 min at 4℃ to prevent non-specific binding of antibodies. Cells were then stained with the fluorescence conjugated primary antibodies for 30 min at 4℃ in the dark. Data were acquired through a BD FACSAria^TM^ III cytometer. All analyses were performed with FlowJo software. Live single macrophages were gated based on forward scatter vs side scatter and confirmed by FVD staining.

**Reverse Transcription and Real Time PCR**

Total RNA were prepared using RNAiso and treated with genomic eraser from Prime Script^TM^ RT reagent kit to remove possible genomic DNA contamination. One microgram of DNA-free total RNA was used to make cDNAs following the instructions of the Prime Script^TM^ RT reagent kit. The real-time PCR procedure was described previously [^37^](#_ENREF_37). The specificity of the amplification products was confirmed by melting curve. The specific gene expression levels were determined by Ct method and individual values for specific genes were normalized to the Ct value of GAPDH. Specific primers used in the real-time PCR to amplify corresponding mRNAs are as follows: IL-6 Forward: TAGTCCTTCCTACCCCAATTTCC; IL-6 Reverse: TTGGTCCTTAGCCACTCCTTC; OSM Forward: GAACACTGCTCAGTTTGACCC; OSM Reverse: GGCGGATATAGGGCTCCAAG; LIF Forward: ATTTGTGCCCTTACTGCTGCTG; LIF Reverse: GCCAGTTGATTCTTGATCTGGT.

**Subcutaneous Tumor Model**

For the subcutaneous tumor model, female C57BL/6J mice were anesthetized with 1.25% tribromoethanol and shaved with an electric razor. Mice were randomly divided into five groups: (1) Vehicle-LLC; (2) LLC-GM-BMDM; (3) LLC-M-BMDM; (4) LLC-M-BMDM-SC144 (5 mg/kg i.p.); (5) Vehicle-LLC-SC144 (5 mg/kg i.p.). Eight mice were placed in group (1) and six in each of the other groups. For LLC group, 5 × 10^5^ *in vitro*-expanded LLC cells were inoculated on the right flanks of mice. For LLC-GM-BMDM group, each mouse was injected with 3 × 10^6^ *in vitro* differentiated GM-BMDMs and 5 × 10^5^ LLC cells. For LLC-M-BMDM and LLC-M-BMDM-SC144 (5 mg/kg i.p.) groups, each mouse was given 2 × 10^6^ M-BMDMs and 5 × 10^5^ LLC cells. One day after inoculation, for LLC-M-BMDM-SC144 (5 mg/kg i.p.) and Vehicle-LLC-SC144 (5 mg/kg i.p.) groups, each mouse received 5 mg/kg SC144 intraperitoneally for 3 consecutive days. All mice were allowed freely access to food, water and activity for 2 weeks. Mice were then sacrificed with CO_2_, and the tumors surgically dissected and weighed. The tumor volume and body weights of the mice were monitored over the experiment period.

**Statistical Analysis**

All the histograms and line charts were made by GraphPad Prism 8. Results were graphed as mean ± SD. Statistical significance was calculated by one-way or two-way ANOVA for comparisons. Significant differences are indicated as * *p* <0.05.
